# Immune Reconstitution Inflammatory Syndrome Mimicking Progressive Multifocal Leucoencephalopathy in a Multiple Sclerosis Patient Treated With Natalizumab: A Case Report and Review of the Literature

**DOI:** 10.14740/jocmr1888w

**Published:** 2014-10-16

**Authors:** Maria-Eleptheria Evangelopoulos, Vasilios Koutoulidis, Kostas Kilidireas, Dimitrios-Stergios Evangelopoulos, Georgios Nakas, Elisabeth Andreadou, Lia-Angela Moulopoulos

**Affiliations:** a Demyelinating Diseases Unit, Department of Neurology, University of Athens, Eginition Hospital, Athens, Greece; b Department of Radiology, University of Athens, Areteion Hospital, Athens, Greece; c The 3rd Department of Orthopaedic Surgery, University of Athens, KAT Hospital, Athens, Greece

**Keywords:** Immune Reconstitution Inflammatory Syndrome, Leucoencephalopathy, Sclerosis, Natalizumab

## Abstract

Natalizumab (NTM) represents an effective drug for the treatment of relapsing-remitting multiple sclerosis (RRMS). Progressive multifocal leucoencephalopathy (PML) is a potential life-threatening complication of NTM treatment. A close follow-up and MRI monitoring of patients under NTM are required to avoid such devastating complications. The case of a 47-year-old woman with RRMS (EDSS 1.5) treated with NTM for 44 months is reported. The patient had a relapse with mild cerebellar symptomatology and visual complaints. MRI revealed a new area of abnormal signal intensity in the subcortical white matter of the right parietal lobe with mild peripheral enhancement. Visual fields showed scotomata mostly of the left eye. NTM was discontinued. JC virus (JCV) polymerase chain reaction (PCR) in cerebrospinal fluid was negative. The patient received IV corticosteroids for 5 days and then monthly for 2 months with subsequent clinical and MRI improvement. On month 4, she presented with a new relapse with severe ataxia, mild behavioral change, increase of cerebellar symptoms and internuclear opthalmoplegia (EDSS 3.5). MRI showed reappearance of the right parietal lobe lesion, with decreased size and less pronounced contrast enhancement. A new 2-cm lesion was noted in the left cerebellar hemisphere with a speckled pattern of contrast enhancement. JCV PCR was negative and the patient was treated with IV corticosteroids. On month 12, she demonstrated clinical and MRI improvement. Although initially PML was highly suspected in this patient, the clinical and MRI findings were supportive of the presence of immune reconstitution inflammatory syndrome (IRIS).

## Introduction

Natalizumab (NTM), a monoclonal antibody against a4 integrin, represents a second-line therapy for relapsing-remitting multiple sclerosis (RRMS), significantly reducing clinical relapses and disease progression [1].

Progressive multifocal leucoencephalopathy (PML) is an opportunistic brain infection caused by reactivation of latent JC virus (JCV) infection of oligodendrocytes and subsequent demyelination of the central nervous system (CNS) [2]. Although it is mainly observed in immunocompromised patients, PML can be a potential complication of NTM treatment. This monoclonal antibody inhibits specific immune functions such as cell migration across the blood-brain barrier and may result in a compromised selected immune surveillance [2].

In the presence of new neurological symptoms in NTM-treated patients, PML needs to be excluded. However, the distinction between MS relapse, PML and immune reconstitution inflammatory syndrome (IRIS) may not be clear at symptoms onset. Clinical history and neurological examination as well as MRI scans and cerebrospinal fluid (CSF) analysis lead to the diagnosis of PML in most cases [3]. Early diagnosis of PML with subsequent NTM discontinuation is essential in limiting brain damage and is associated with a better prognosis [4].

## Case Report

A 47-year-old woman with an 11-year history of RRMS was under NTM treatment for 44 months.

The patient was initially treated with interferon for 3 years and switched to NTM treatment due to two significant clinical relapses. It is of note that one of her relapses consisted of Wernicke’s aphasia and cerebellar symptomatology and responded well to corticosteroid treatment. During NTM treatment, she remained relapse free (EDSS 1.5) and underwent regular MRI monitoring every 6 months showing no new lesions.

**Figure 1:**
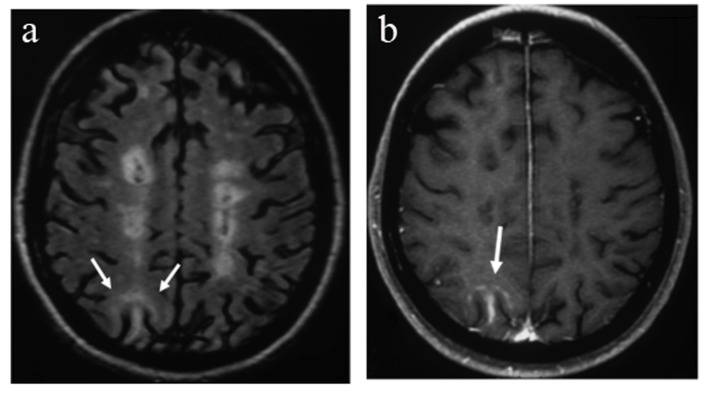
MRI on initial relapse reveals a new lesion in the subcortical white matter of the right parietal lobe with high signal on the FLAIR sequence (arrows, a) and mild contrast enhancement (arrow, b).

**Figure 2:**
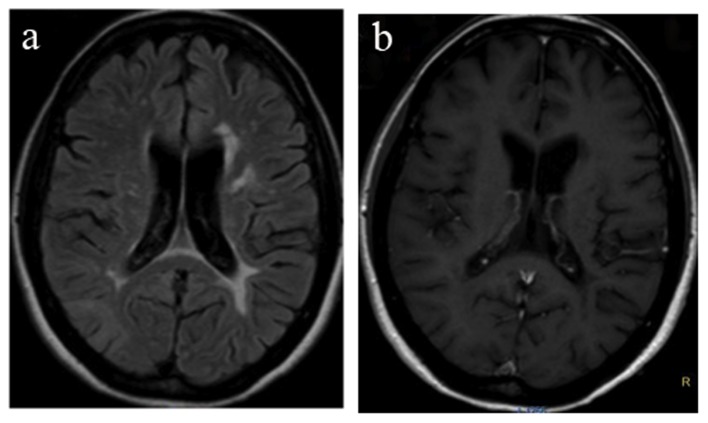
MRI 2 months after demonstrating a resolution of the lesion of the right parietal lobe (a) and absence of gadolinium enhancement (b).

After NTM treatment for 44 months, she presented with a relapse with mild cerebellar symptomatology and visual complaints. She was found positive for JCV antibodies in serum while antibodies against NTM were negative. MRI showed a new area of abnormal signal intensity in the subcortical white matter of the right parietal lobe with mild peripheral enhancement (Fig. 1a, b). Visual fields showed scotomata mostly of the left eye. NTM treatment was discontinued. Although the patient had no specific clinical signs for PML, brain MRI was suspicious and lumbar puncture was performed. The CSF biochemistry and cell counts were normal. Polymerase chain reaction (PCR) for the detection of JCV DNA in CSF was negative. The patient received IV corticosteroids for 5 days and then monthly for 2 months with subsequent clinical and MRI improvement (Fig. 2a, b).

**Figure 3:**
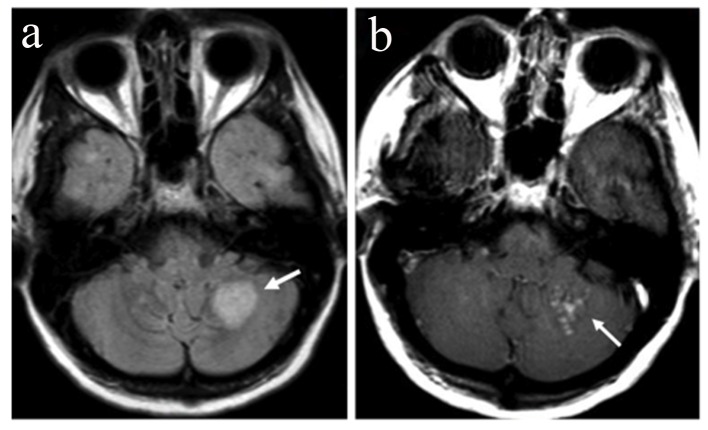
On month 4, MRI demonstrates a new 2-cm lesion in the left cerebellar hemisphere with high signal on FLAIR (arrow, a) and a speckled pattern of contrast enhancement on the post-gadolinium image (arrow, b).

**Figure 4:**
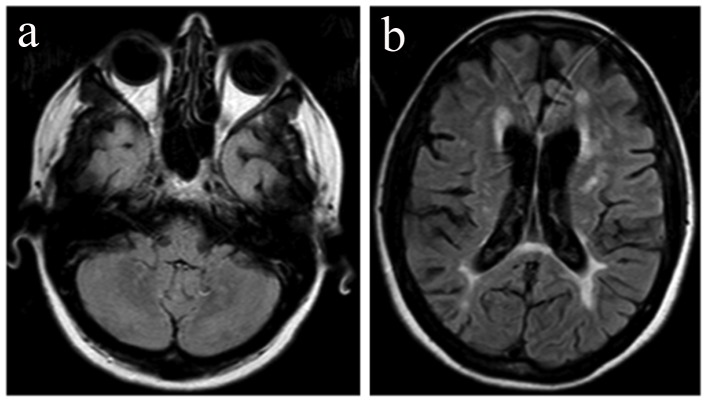
On a follow-up MRI scan 12 months after the initial relapse, FLAIR images reveal complete resolution of the signal abnormalities in the left cerebellar hemisphere (a) and in the right parietal lobe (b).

On month 4, she presented with a new relapse with severe ataxia, mild behavioral change, increase of cerebellar symptoms and internuclear opthalmoplegia (EDSS 3.5). MRI showed reappearance of the right parietal lobe lesion, albeit with decreased size and less pronounced contrast enhancement. Additionally a new 2-cm lesion was found in the left cerebellar hemisphere. In post-contrast images, the cerebellar lesion showed enhancement with a speckled pattern, consisting of multiple coalescing punctate foci (Fig. 3a, b). In addition, new small foci of enhancement were present at multiple sites of the subcortical white matter. Lumbar puncture was repeated and JCV PCR was again negative. The patient was treated with IV corticosteroids for 5 days. On month 6, 8, 10 and 12 follow-up, the patient showed clinical and MRI improvement (Fig. 4a, b) but still remained under monthly IV corticosteroid treatment.

## Discussion

We present a patient with RRMS under NTM treatment for 44 months who presented with a relapse and MRI findings suspicious of PML were finally diagnosed as a non-PML-IRIS.

IRIS is defined as paradoxical worsening of the neurological condition during immune recovery in a patient with CNS infection such as HIV [4]. Lately, it is described as a complication of PML in MS patients treated with NTM [2]. NTM prevents T lymphocytes binding to endothelial cells and trafficking across blood-brain barrier, leading to compartmental immune suppression [4]. It is proposed that cessation of monoclonal antibody therapy in PML re-establishes immune surveillance for JCV-infected cells in the CNS and leads to clinically apparent inflammatory responses in the brain [5]. As MS patients have an intact immune system, removal of NTM results in an influx of lymphocytes in the CNS and the development of severe IRIS [4]. Literature data support that IRS may be an inflammatory immune response directed against myelin antigens [6].

The present case underlines the difficulty to differentiate PML from IRIS at the onset of neurological symptoms. Although there was only a mild change in patient’s neurological status, MRI scan was suspicious for PML. The repeated PCR for JCV at different time intervals as well as the clinical improvement of the patient’s clinical status excludes the diagnosis of PML with low JCV DNA copies [7].

However, clinical vigilance of patients under prolonged NTM is necessary to access any change in neurological status since in the presymptomatic phase of PML, the presentation may be atypical [7]. A follow-up from the same neurologists might permit the evaluation of even slight changes of patient’s clinical status, suspicious of PML.

Metz et al studied the histopathological and MRI findings of IRIS in a series of five MS patients with NTM associated PML after cessation of therapy. They reported that one of the enhancement patterns compatible with IRIS is the linear and speckled enhancement of the gray-white matter junction or within the gray matter [8]. A similar speckled pattern of enhancement was encountered in the cerebellar lesion that our patient developed at the time of her second severe relapse.

On the other hand, contrast enhancement in MRI was reported in 12 of 28 (43%) of patients with PML, suggesting that contrast-enhancing lesions cannot favor MS exacerbation and exclude PML without further investigation [3].

IRIS has been described in association with PML in MS patients under NTM treatment [9]. Two different forms of IRIS have been described in association with PML. Immunologic restoration of immune system after NTM cessation may lead to the development of IRIS within 3 months. It is proposed that in early PML-RIS with Gd enhancement, auto-reactive T lymphocytes enter CNS through defective blood-brain barrier and kill JCV-infected oligodendrocytes leading to release of viral DNA into the CSF [9].

The present case shows that IRIS may present in the absence of PML and underlines the necessity of careful clinical and MRI monitoring of NTM-treated patients to diagnose a relapse from PML. In spite the increased risk of developing a dramatic clinical and radiological worsening after NTM discontinuation, our patient showed a good outcome after regular corticosteroid treatment for 10 months.
